# Precise control of microfluidic flow conditions is critical for harnessing the in vitro transfection capability of pDNA-loaded lipid-Eudragit nanoparticles

**DOI:** 10.1007/s13346-024-01523-y

**Published:** 2024-02-12

**Authors:** Diviya Santhanes, Huiming Zhang, Alex Wilkins, Robert John Aitken, Anne-Louise Gannon, Mingtao Liang

**Affiliations:** 1 School of Biomedical Sciences and Pharmacy, University of Newcastle, Callaghan, NSW 2308 Australia; 2 Electron Microscopy and X-Ray Unit, Research and Innovation Division, University of Newcastle, Callaghan, NSW 2308 Australia; 3 School of Environmental and Life Sciences, University of Newcastle, Callaghan, NSW 2308 Australia

**Keywords:** Microfluidics, Eudragit, Lipid-polymer hybrid nanoparticles, Flow rate ratio, Gene transfection, Cytotoxicity

## Abstract

Microfluidics is widely regarded as a leading technology for industrial-scale manufacture of multicomponent, gene-based nanomedicines in a reproducible manner. Yet, very few investigations detail the impact of flow conditions on the biological performance of the product, particularly biocompatibility and therapeutic efficiency. Herein, this study investigated the engineering of a novel lipid-Eudragit hybrid nanoparticle in a bifurcating microfluidics micromixer for plasmid DNA (pDNA) delivery. Nanoparticles of ~150 nm in size, with uniform polydispersity index (PDI = 0.2) and ξ-potential of 5–11 mV were formed across flow rate ratios (FRR, aqueous to organic phase) of 3:1 and 5:1, respectively. The hybrid nanoparticles maintained colloidal stability and structural integrity of loaded pDNA following recovery by ultracentrifugation. Importantly, in vitro testing in human embryonic kidney cell line (HEK293T) revealed significant differences in biocompatibility and transfection efficiency (TE). Lipid-Eudragit nanoparticles produced at FRR 3:1 displayed high cellular toxicity (0–30% viability), compared with nanoparticles prepared at FRR 5:1 (50–100% viability). Red fluorescent protein (RFP) expression was sustained for 24–72 h following exposure of cells to nanoparticles, indicating controlled release of pDNA and trafficking to the nucleus. Nanoparticles produced at FRR 5:1 resulted in markedly higher TE (12%) compared with those prepared at FRR 3:1 (2%). Notably, nanoparticles produced using the bench-scale nanoprecipitation method resulted in lower biocompatibility (30–90%) but higher RFP expression (25–38%). These findings emphasize the need for in-depth analysis of the effect of formulation and flow conditions on the physicochemical and biological performance of gene nanomedicines when transitioning from bench to clinic.

**Graphical abstract:**

Transitioning lipid-Eudragit hybrid nanoparticles from bench-scale nanoprecipitation to industrial-scale microfluidics

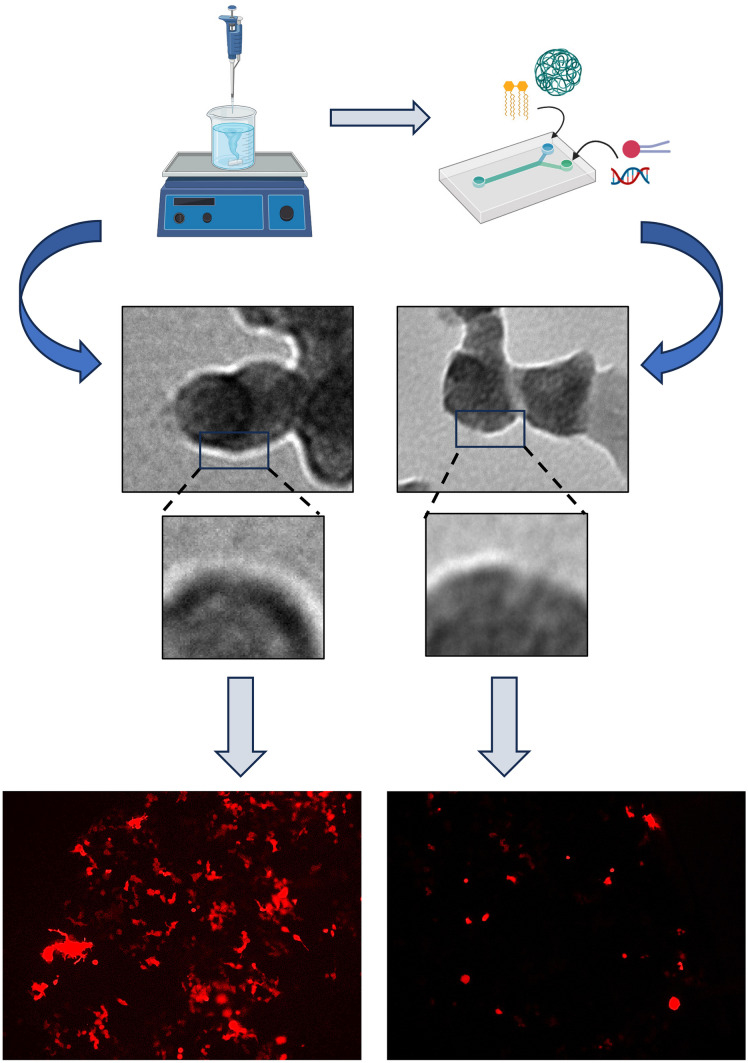

## Introduction

Gene medicines featuring nanoparticle delivery systems continue to offer a powerful strategy for treating diverse medical conditions [1]. Their ability to mediate site-specific delivery of nucleic acids including short-interfering RNA (siRNA), messenger RNA (mRNA), and plasmid DNA (pDNA) has been demonstrated in a multitude of studies targeting genetic disorders, neurodegenerative conditions, and vaccination [2–4]. Indeed, the availability of several nucleic acid-based nanomedicines, *e.g.*, Onpattro^®^, Givlaari^®^, and mRNA-1273 in the global market affirms their clinical utility [5]. However, progress in developing gene therapeutics overall has been severely restricted by a number of barriers, both nanotechnological and physiological. A major limitation is the production of nanoparticles with reproducible properties in sufficient quantities to meet clinical requirements [6]. It is also widely acknowledged that improvements in gene transfection efficiency, biocompatibility, and long-term storage stability of experimental nanomedicines pose severe challenges which are hindering clinical translation.

The nanoparticle carriers for gene medicines have been produced from a wide range of materials including lipids, polymers, exosomes, and inorganic components including gold and graphene [7]. One emerging technology combines the advantages of individual lipid and polymer carriers in a lipid-polymer hybrid nanoparticle format. A polymer core promotes high structural stability to overcome a major drawback of lipid carriers, while a lipid shell confers improved biocompatibility [8]. The hybrid nanoparticles are often produced using lipid-polyethylene glycol (PEG) surfactants to achieve steric stability of nanosuspensions [9]. The resulting modification or PEGylation of the nanoparticle surface may also confer a ‘stealth’ characteristic following intravenous (IV) administration by impeding phagocytic uptake by cells of the reticuloendothelial system (RES) [10].

Lipid-polymer hybrid nanoparticles have been developed predominantly at benchtop-scale using double emulsion and nanoprecipitation methods and have been shown capable of nucleic acid delivery in vitro and in vivo [11–13]. For example, Su et al*.* [11] produced lipid-polymer nanoparticles based on poly-β-amino ester (PBAE) for intranasal delivery of luciferase mRNA. Yang et al*.* [12] prepared polymer blend nanoparticles using poly-lactic-*co*-glycolic acid (PLGA) and poly-L-lysine (PLL) for DNA vaccination against Ebola infection. While these and other studies [2, 13] have established the potential of experimental gene nanomedicines based on hybrid nanoparticles, clinical translation is impeded due to the complex, multi-step, and discontinuous production techniques involved.

Microfluidics, defined as the study of fluid flow in micron-sized channels, offers an automated platform for continuous nanomedicine production at bench and industrial-scale [14]. Uniform and rapid mixing of fluids in a controlled manner leads to precise nanoparticle size, high batch-to-batch reproducibility, and large-scale manufacture [15–20]. Nanoparticles have also been produced from a wide variety of materials of organic (emulsions, lipid-based, and polymer conjugates) and inorganic nature (quantum dots, iron-oxide, and silica nanostructures) [18, 21]. Although the wealth of published work has demonstrated the promise of microfluidics for production of nanomedicines, research has predominantly investigated the effect of flow conditions on nanoparticle size, polydispersity index (PDI), and efficiency of drug/gene encapsulation [20, 22–26]. Reports of the influence of flow conditions on the biological performance of experimental nanomedicines are extremely rare in the literature, although such investigations are of fundamental importance for identifying potential candidates for clinical translation. Only one report by Quagliarini et al*.* [27] on pDNA-loaded cationic lipid nanoparticles provided a correlation between microfluidic flow conditions (TFR = 2 mL/min and 8 mL/min) and luciferase expression in vitro.

**Fig. 1:**
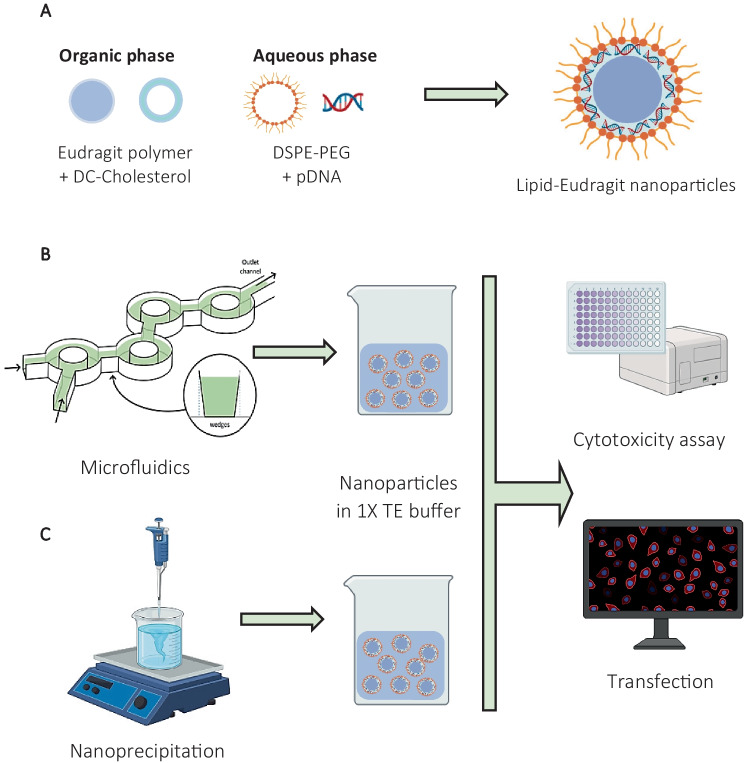
**A)** Schematic depicting components for formulation of lipid-Eudragit hybrid nanoparticles; **B)** Continuous manufacture using bifurcating microfluidics mixer; **C)** Discontinuous, bench-scale production using nanoprecipitation. Images reproduced with permission from Biorender®.com and [28, 29]. Copyright 2022, Elsevier Group, Inc. and Copyright 2022, Nature Publishing Group, Inc

Thus, the aim of the present study was to explore the effect of microfluidic conditions, principally flow rate ratio (FRR) on the physicochemical characteristics (size and PDI), and biological performance (biocompatibility and transfection efficiency) of novel lipid-Eudragit hybrid nanoparticles (Fig. 1). Cationic, pH-sensitive Eudragit E100 was selected as the core polymer as an alternative to the commonly used poly-lactic-*co*-glycolic acid (PLGA) (Fig. 1A). DC-Cholesterol, a cationic lipid, was included to enhance the DNA transfection capability of Eudragit [30], which is generally limited to 12% [31, 32]. The nanoparticle surface was modified by lipid-polyethylene glycol (DSPE-PEG) surfactant to provide steric stabilisation of the nanosuspensions [9].

pDNA-loaded samples were produced using a bifurcating (toroidal) micromixer (Fig. 1B) and were expected to exhibit a typical core/shell structure composed of the hydrophobic cationic Eudragit E100 as the core matrix, coated with a lipid membrane of DC-Cholesterol/DSPE-PEG (Fig. 1A). The microfluidic fabricated nanoparticles were compared with those produced using nanoprecipitation, a common benchtop protocol for preparation of lipid-polymer hybrid nanoparticles (Fig. 1C).

## Materials and methods

### Materials

Cationic Eudragit E100 polymer (*M*_w_: 47, 000 g/mol) was kindly provided by Evonik Röhm Gmbh (Darmstadt, Germany) and used as supplied. 3β-[N-(N′, N′-Dimethylaminoethane)-carbamoyl] cholesterol hydrochloride (DC-Cholesterol), *M*_w_: 537.26 Da was procured from Santa Cruz Biotechnology, Texas, USA. Tris-EDTA (TE) buffer solution (10 mM Tris, 1 mM EDTA, pH=8) was obtained from Sigma-Aldrich, Macquarie Park, Australia. 1,2-Distearoyl-sn-glycero-3-phosphoethanolamine-N-methoxy polyethylene glycol (DSPE-PEG, PEG *M*_w_=2000 Da) was sourced from Laysan Bio. Inc, Alabama, USA. Quant-iT™ PicoGreen^®^ dsDNA Assay kit and SYBR™ Safe DNA Gel Stain were obtained from Molecular Probes, Inc., Invitrogen, Oregon, USA. Commercial transfection reagent Lipofectamine 3000™ was purchased from Invitrogen, Life Technologies, Carlsbad, CA, USA.

Human embryonic kidney (HEK293T) cell line was procured from American Type Culture Collection (ATCC), Virginia, USA. Supplemental components Dulbecco’s Modified Eagle’s Medium (DMEM) and 200 mM L-glutamine were also products from ATCC (Virginia, USA). Heat-inactivated foetal bovine serum (FBS) and 1% penicillin-streptomycin antibiotic (10,000 units/mL of penicillin and 10,000 µg/mL of streptomycin) were acquired from Cell Sera, Australia and Gibco, New York, USA, respectively. Calcium and magnesium (Ca^2+^ and Mg^2+^) free phosphate-buffered saline (PBS) was obtained from Sigma-Aldrich, Missouri, USA. Heparin sodium salt (157 U.S.P units per mg of solid, from porcine intestinal mucosa) was sourced from Sigma-Aldrich, St. Louis, Missouri, USA. All organic solvents used were of analytical grade.

### Plasmid DNA (pDNA) extraction and purification

The plasmid pLenti-N-tRFP (7 kbp) lentiviral vector with N-terminal tRFP tag (encoding for red fluorescent protein, RFP) was sourced from OriGene Technologies (Rockville, Maryland, USA). pDNA extraction and purification was conducted as reported previously [28]. In brief, pDNA was transformed in *E. coli* cells, propagated in Luria-Bertani (LB) broth, and purified as outlined by the manufacturer’s protocol (QIAGEN^®^ Plasmid Maxi Kit, Hilden, Germany). The concentration was determined using UV-visible spectrophotometry at absorbance wavelength ratios (260:280 nm) of 1.8 or greater (Nanodrop Lite, Thermo Fisher Scientific, Delaware, USA). Purified pDNA was dissolved in 1X TE buffer and stored at –30 °C before use.

### Production of lipid-Eudragit hybrid nanoparticles using microfluidics

The bifurcating (toroidal) micromixer (NxGen cartridge, Precision Nanosystems Inc. Vancouver, Canada) was employed for single-step, continuous preparation of unloaded and pDNA-loaded hybrid nanoparticles. The organic phase containing polymer (Eudragit E100) and lipid (DC-Cholesterol) was prepared by first dissolving each component separately in methanol according to the starting weights and volumes listed in Table 1. The solutions were subsequently combined to provide final concentrations of polymer and lipid of 10 mg/mL and 1 mg/mL, respectively. The aqueous phase consisted of 1 mg/mL DSPE-PEG and 8.6 µg/mL RFP pDNA in 1X TE buffer, optimized previously using PLGA-based hybrid nanoparticles [28]. In the case of unloaded nanoparticles, only DSPE-PEG surfactant was present in the aqueous phase.

**Table 1:** Formulation parameters for microfluidic production of lipid-Eudragit hybrid nanoparticles

**Organic phase**					**Aqueous phase**			**Microfluidic conditions**
**Eudragit E100 (mg)**	**Polymer soln. volume (mL)**	**DC-Cholesterol (mg)**	**Lipid soln. volume (mL)**	**Total volume (mL)**	**DSPE-PEG (mg)**	**RFP pDNA (µg)**	**Total volume (mL)**	**FRR (aqueous to organic phase)**
15	0.9	1.5	0.6	1.5	4.5	38.7	4.5	3:1
10	0.6	1	0.4	1	5	43	5	5:1

The organic and aqueous phases were loaded separately into single-use, Luer-Lok™ polypropylene syringes (Becton Dickinson, Singapore) and delivered to the mixing section of the micromixer cartridge chip via separate channels using syringe pumps (Pump 11 Elite Syringe Pump, Harvard Apparatus, Massachusetts, USA). Flow conditions were selected based on previous studies involving lipid-PLGA nanoparticles [28]. Two flow rate ratios (FRR) of aqueous to organic phases were investigated, namely, 3:1 and 5:1 mL/min (Table 1) with total flow rate (TFR) kept constant at 6 mL/min.

Several studies have indicated that microfluidic FRR impacts the size of polymer, hybrid, and liposomal nanoparticles, whereas the effect of TFR is minor [33–35]. In particular, Gdowski et al*.* [34] reported that using a TFR of 6–12 mL/min at polymer concentrations 10–15 mg/mL did not result in a significant change in lipid-polymer hybrid nanoparticle size. The present study employed a constant TFR of 6 mL/min and polymer concentration of 10 mg/mL (Table 1); thus, no significant change in nanoparticle size was anticipated.

### Production of lipid-Eudragit hybrid nanoparticles using nanoprecipitation

Lipid-Eudragit nanoparticles were produced using a facile one-step nanoprecipitation protocol, as previously described [28]. In brief, 10 mg of Eudragit E100 polymer was dissolved in 0.6 mL methanol and 1 mg DC-Cholesterol was dissolved separately in 0.4 mL of methanol. The solutions were combined to form a clear organic phase comprising polymer and lipid of concentration 10 mg/mL and 1 mg/mL, respectively. The aqueous phase (5 mL) contained 1 mg/mL DSPE-PEG and 8.6 µg/mL RFP pDNA in 1X TE buffer (pH = 8). The organic phase was added jet-wise using a micropipette to the aqueous phase featuring a central vortex, produced by magnetic stirring at 700 rpm (IKA RT-10 Magnetic Stirrer, Staufen, Germany).

### Ultracentrifugation procedure

For both microfluidics and nanoprecipitation-based production, unloaded and pDNA-loaded nanoparticles formed spontaneously upon combining the organic and aqueous phases and were collected in 10 mL glass vials. The nanoparticle suspensions underwent solvent evaporation in a fume hood for 24 h under magnetic stirring conditions at 700 rpm (IKA RT-10 Magnetic Stirrer, Staufen, Germany). Nanoparticle recovery and washing was carried out using ultracentrifugation at 100, 000 *g* for 10 min twice at 4°C (Optima Max-XP, Beckman Coulter, Indianapolis, USA). Unloaded and pDNA-loaded nanosuspensions (3 mL) were subjected to a first wash and the supernatant was kept aside for pDNA quantification purposes (section “Determination of pDNA loading in nanoparticles”). The pellet was resuspended in Milli-Q Ultrapure water and washed for a second time. The final pellet obtained was resuspended in 1X TE buffer and the nanosuspensions were stored at 4°C prior to physicochemical and biological analysis.

### Measurement of nanoparticle size and surface charge

The hydrodynamic diameter and polydispersity index (PDI) of nanoparticles were determined using dynamic light scattering (DLS, Zetasizer Pro, Malvern Panalytical, Worcestershire, UK). Nanosuspension samples were diluted in deionised water at 25°C and three measurements were obtained for each sample at a backscattering angle of 173°. The ζ-potential of nanoparticles (undiluted, suspended in 1X TE buffer) was analysed using Electrophoretic Light Scattering (ELS) with M3-PALS (Zetasizer Pro, Malvern, UK) following washing since the presence of excipients can affect the surface charge.

### High-resolution transmission electron microscopy (TEM)

Morphological examination of recovered nanoparticles was carried out using a JEOL JEM-2100 high resolution transmission electron microscope (HRTEM, JEOL, Tokyo, Japan) at an accelerating voltage of 120 kV. Unloaded and pDNA-loaded samples were prepared by depositing 30 μL of nanoparticle suspension (1.5 mg/mL) onto a 300-mesh carbon/formvar coated glow-discharged TEM grid. Excess fluid was blotted with a filter paper after 1 h and the grids were negatively stained with 2% (w/v) uranyl acetate for 10 min. Stained grids were washed three times in Milli-Q water (Merck-Millipore, USA) by dipping the grids in 40 μL water droplets and air-dried overnight prior to imaging.

### Determination of pDNA loading in nanoparticles

The encapsulation efficiency (EE, %) of RFP pDNA in nanoparticles was determined using the Quant- iT™ PicoGreen^®^ dsDNA Assay kit (Invitrogen, Oregon, USA) according to manufacturer’s recommendations. A standard calibration curve was constructed by dissolving pDNA in the supernatant obtained from unloaded nanoparticles (concentration=0.001–1 µg/mL) to offset interference effects [28]. Fluorescence readings were recorded using fluorescence spectrophotometry (FLUOstar Optima plate reader, BMG Labtech, Ortenburg, Germany), at an excitation wavelength of 485 nm and emission wavelength 520 nm. Supernatants obtained following the first ultracentrifugation wash for pDNA-loaded samples were assayed to provide measurements of free, unencapsulated pDNA. The weight of pDNA loaded in the nanoparticles was obtained by subtraction and the encapsulation efficiency was calculated using Eq. 1. pDNA loading of the nanoparticles (% w/w) was determined using Eq. 2 following freeze-drying of nanosuspension (1.5 mL) at –40 °C for 24 h (Dyna-Vac Freeze Drier, Western Australia, Australia).

1 $${\text{EE}} (\%)=\frac{\text{Weight of pDNA in nanoparticles }(\mathrm{\mu g})\times100\mathrm{\% }}{\text{Starting weight of pDNA used in formulation }(\mathrm{\mu g})}$$

2 $$\text{pDNA loading }(\mathrm{\% w}/{\text{w}}) = \frac{\text{Weight of pDNA in nanoparticles }(\mathrm{\mu g})\times100\mathrm{\%}}{\text{Weight of recovered nanoparticles }(\mathrm{\mu g})}$$

### Structural integrity of pDNA-loaded nanoparticles

Gel electrophoresis was conducted to analyze the structural integrity of pDNA associated with lipid-Eudragit nanoparticles. Unloaded nanoparticle samples were also analyzed for background correction. Nanoparticles were incubated in equivalent volume (1:1 v/v) of high concentration polyanion heparin sodium (10 mg/mL in 1X TE buffer) for 18 h at 25°C to dissociate bound pDNA from the nanoparticles.

Samples of nanosuspension (15 µL) were loaded in 1% w/v agarose gel (Promega Corporation, Madison, WI, USA). pDNA migration from the nanoparticles was induced in 1X Tris-acetate EDTA (TAE) buffer (40 mM Tris (pH 7.6), 20 mM acetic acid, and 1 mM EDTA) at a voltage of 140 V for 45 min. DNA bands were visualized using SYBR™ Safe DNA Gel Stain (Invitrogen, Oregon, USA) and gel images were captured under UV light using ChemiDoc MP Imaging System (Bio-Rad, California, USA). A 1kb DNA ladder and naked RFP pDNA in 1X TE buffer were run for comparison.

### Cell culture

Human embryonic kidney cell line (HEK293T) was stored, grown and maintained according to manufacturer’s guidelines (American Type Culture Collection, ATCC, Virginia, USA). In brief, cells were grown in high-glucose DMEM, supplemented with 10% heat-inactivated FBS, 1% penicillin-streptomycin antibiotic (100 U/ml penicillin and 100 μg/mL streptomycin) and 1% 2 mM L-Glutamine. Cell culture and biological experiments were maintained in humidified conditions of 37°C and 5% CO_2_/95% O_2_. Cells were sub-cultured twice weekly and were used between passages 3 and 12.

### Cytotoxicity evaluation of nanoparticles

Cell viability following exposure to lipid-Eudragit nanoparticles was investigated using the MTT cell proliferation assay (3-[4,5-dimethylthiazol-2-yl]-2,5-diphenyltetrazolium bromide). HEK293T cells were grown in 96-well plates (flat-bottom) for 24 h at a seeding density of 5,000 cells in 100 µL complete growth medium. The growth medium was removed, and cells were washed with Ca^2+^ and Mg^2+^ free-PBS and replaced with 100 µL of 1% antibiotic treated reduced serum medium (Opti-MEM I, Long Island, NY, USA, Gibco). Nanoparticles were added to cells at the following pDNA doses: 0.02, 0.03, 0.06, 0.13, 0.25, and 0.50 µg/mL. Following incubation at 37°C for 4 h, the nanoparticle-containing medium was removed and replaced with 100 µL of complete growth medium. The metabolic activity of the cells was measured at 24, 48, and 72 h following nanoparticle addition, in line with transfection time points [36], using the MTT labelling reagent.

MTT reagent in PBS (10 µL) was added to each of triplicate sample wells. After incubation for 4 h, the formazan crystals were dissolved in 100 µL of solubilization buffer (10% sodium dodecyl sulphate (SDS) in 0.01 M hydrochloric acid (HCl)) and the plate was incubated at 37°C overnight. Absorbance readings were obtained at 570 nm using a microplate reader (Clariostar PLUS Plate reader, BMG LABTECH Gmbh, Ortenberg, Germany). Cell viability was calculated as a percentage of the mean absorbance readings of nanoparticle-exposed samples relative to the control (untreated) cells. The commercial transfection agent Lipofectamine 3000™ (Invitrogen, Life Technologies, Carlsbad, CA, USA) was used as a positive control according to manufacturer’s instructions (pDNA dose=1 µg/mL), while naked, unformulated DNA was utilized as a negative control. All samples were tested in triplicate.

### Analysis of transfection efficiency

HEK293T cells were seeded in 24-well plates at a density of 10,000 cells per 1 mL of complete growth medium and left to adhere for 48 h to achieve ~60% confluence. Prior to nanoparticle addition, the complete growth medium was removed, and cells were washed with Ca^2+^ and Mg^2+^ free-PBS and replaced with 500 µL of reduced serum medium (1% antibiotic treated, Opti-MEM I). Nanosuspension was added to cells to obtain pDNA doses of 0.02, 0.03, 0.06, 0.13, 0.25, and 0.50 µg/mL respectively. Lipofectamine 3000™ was prepared according to manufacturer’s instructions and used to deliver a pDNA dose=1 µg/mL as a comparison. The cells were incubated with the nanoparticles for 4 h before removal of the medium and replacement with 1 mL of complete growth medium. RFP expression was examined at 24, 48, and 72 h following nanoparticle addition to investigate mechanistic profiling of transfection. RFP fluorescence was captured by cell counting (500–800 cells per time point) using an inverted fluorescence microscope and 20× objective lens (EVOS M5000, Invitrogen, Washington, USA) according to established protocols [37]. Transfection efficiency (TE) was calculated as the number of red fluorescent cells divided by the total cell number and reported as a percentage.

### Statistics

All data were expressed as mean ± standard deviation of three independent nanoparticle batches. Statistically significant differences were assessed by one-way analysis of variance (ANOVA) using GraphPad Prism, version 9.0 (GraphPad, La Jolla, CA, USA), with nanoprecipitation-prepared samples assigned as the control group. A *P* value < 0.05 was considered to be statistically significant and significance was denoted as follows: ^*^P: 0.01–0.05, ^**^P: 0.001–0.01, and ^***^P: 0.0001–0.001.

## Results and discussion

### Microfluidic formation of lipid-Eudragit hybrid nanoparticles

Several variations of lipid-polymer hybrid nanoparticles have been produced using microfluidics for nucleic acid delivery but are principally based on poly-lactic-*co*-glycolic acid (PLGA) [24, 26]. Cerda et al*.* [23] utilized a glass-capillary microfluidic co-flow device to fabricate siRNA-loaded cationic lipid cKK-E12-PLGA nanoparticles, which achieved more efficient green fluorescent protein (GFP) gene silencing (~2%) than Lipofectamine (~60%) in RAW 264.7 cells. Similarly, Meyer et al*.* [24] reported luciferase mRNA delivery in vivo, primarily to the liver using D-Lin-MC3-DMA-PLGA nanoparticles produced using microfluidics. However, both studies involved multi-step synthesis, whereby a lipid shell or nucleic acids were adsorbed onto pre-formed nanoparticles [23, 24].

The present study investigated single-stage formation of pDNA-loaded lipid-Eudragit hybrid nanoparticles using the microfluidics toroidal micromixer platform. Nanoparticles are considered to form on combining the organic and aqueous phases by precipitation of the polymer and cationic lipid into a core matrix, followed by surface assembly of the negatively charged lipid-PEG surfactant and pDNA via hydrophobic and electrostatic interactions [38]. The formulation of the hybrid nanoparticles (Table 1) is based on keeping the concentration of excipients in organic and aqueous phases constant, i.e., polymer (10 mg/mL), lipid (1 mg/mL), DSPE-PEG (1 mg/mL), pDNA (8.6 µg/mL), and varying FRR (aqueous phase volume to organic phase volume). Nanoprecipitation involved adding 1 mL organic phase (polymer conc. 10 mg/mL, lipid conc. 1 mg/mL) to 5 mL aqueous phase (DSPE-PEG conc. 1 mg/mL, pDNA conc. 8.6 µg/mL). The excipient concentration of the aqueous and organic phases was equivalent to the FRR 5:1 microfluidics formulation.

**Fig. 2:**
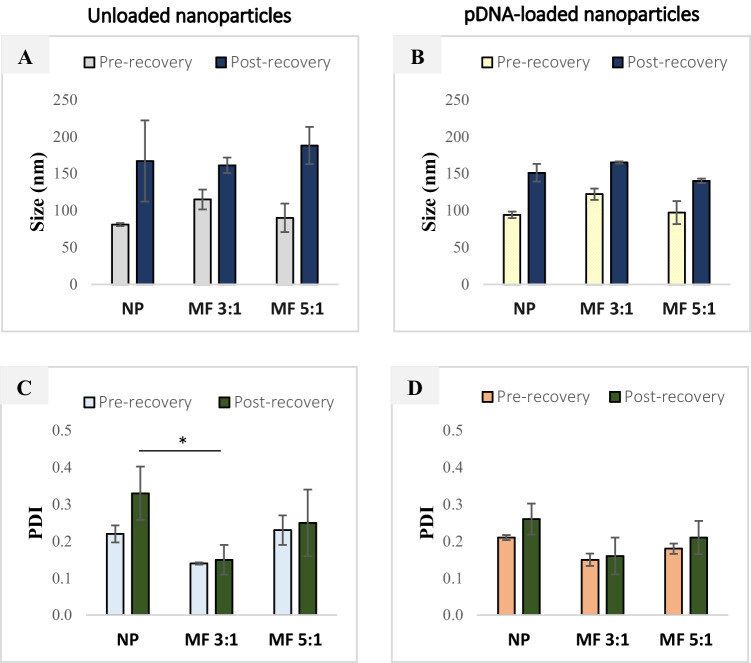
The size and polydispersity index (PDI) of lipid-Eudragit hybrid nanoparticles pre- and post-recovery by ultracentrifugation. Significance was determined using one-way ANOVA (*P: 0.01–0.05). Abbreviations: NP: nanoprecipitation; MF 3:1: microfluidics at FRR 3:1; MF 5:1: microfluidics at FRR 5:1

The resulting size and PDI profile of hybrid nanoparticles produced using microfluidics and nanoprecipitation, respectively, are displayed in Fig. 2. Unloaded nanoparticles, formed by nanoprecipitation exhibited the smallest size of 81 nm, prior to recovery by ultracentrifugation, compared with 90 nm and 115 nm for nanoparticles produced using microfluidics at FRR 5:1 and FRR 3:1, respectively (Fig. 2A). The presence of pDNA in the aqueous phase resulted in relatively small increases in size to 94–122 nm (Fig. 2B). The nanoparticle size distribution remained highly monodisperse (PDI values < 0.2) irrespective of pDNA loading (Fig. 2C, D). The similarity of nanoparticle size and PDI between microfluidic and nanoprecipitation samples is unsurprising and has been widely reported in the literature [25, 39]. A study by Streck et al*.* [39], for example, described PLGA nanoparticle surface-modified with cell-penetrating peptides of similar sizes (150–200 nm) when produced using both nanoprecipitation and microfluidics.

A major factor restraining development of gene medicines is related to concerns over the poor structural stability of nanoparticles [40]. We therefore employed ultracentrifugation for nanoparticle recovery and washing purposes to ensure isolation of nanoparticles from free pDNA and simultaneously to provide information on the physical strength of the nanoparticles, their redispersion capability, and colloidal stability of the suspensions through particle size measurements [23, 41].

While high-speed recovery by ultracentrifugation resulted in nanoparticle size increase, the formulations remained below 200 nm and were of monodisperse distribution (PDI ≤ 0.3) (Fig. 2). Unloaded nanoparticles exhibited a final size range of 161–188 nm compared with 151–165 nm for pDNA-loaded formulations (Fig. 2A, B). However, there was no statistically significant difference in mean size for nanoprecipitation and microfluidics-produced nanoparticles. The PDI of unloaded and pDNA-loaded nanoparticles post-recovery was generally confined to below 0.3 for both microfluidics and nanoprecipitation (Fig. 2C, D). Only unloaded nanoparticles displayed a statistically significant increase above 0.3 (Fig. 2C).

Both microfluidic and nanoprecipitation methods resulted in nanoparticles with small positive surface charge (Table 2). However, the ζ-potential of FRR 3:1 nanoparticles was significantly higher (~2-fold) (10.4 mV) than FRR 5:1 and nanoprecipitation samples (5.7 mV) (^***^P < 0.001, one-way ANOVA). This behavior indicates a lower presence of anionic DSPE-PEG surfactant and/or higher presence of cationic Eudragit and lipid components at the nanoparticle surface, directly reflecting the formulation composition (Table 1).

**Table 2:** ξ-potential (mV) of lipid-Eudragit nanoparticles produced using nanoprecipitation and microfluidics (*n* = *3*)

**Nanoparticle**	**Nanoprecipitation**	**Microfluidic FRR**	
		3:1	5:1
Unloaded	5.1 ± 0.0	11.1 ± 0.6	6.0 ± 0.5
pDNA-loaded	4.8 ± 0.8	10.4 ± 2.8	5.7 ± 0.6

### Nanoparticle morphology

**Fig. 3:**
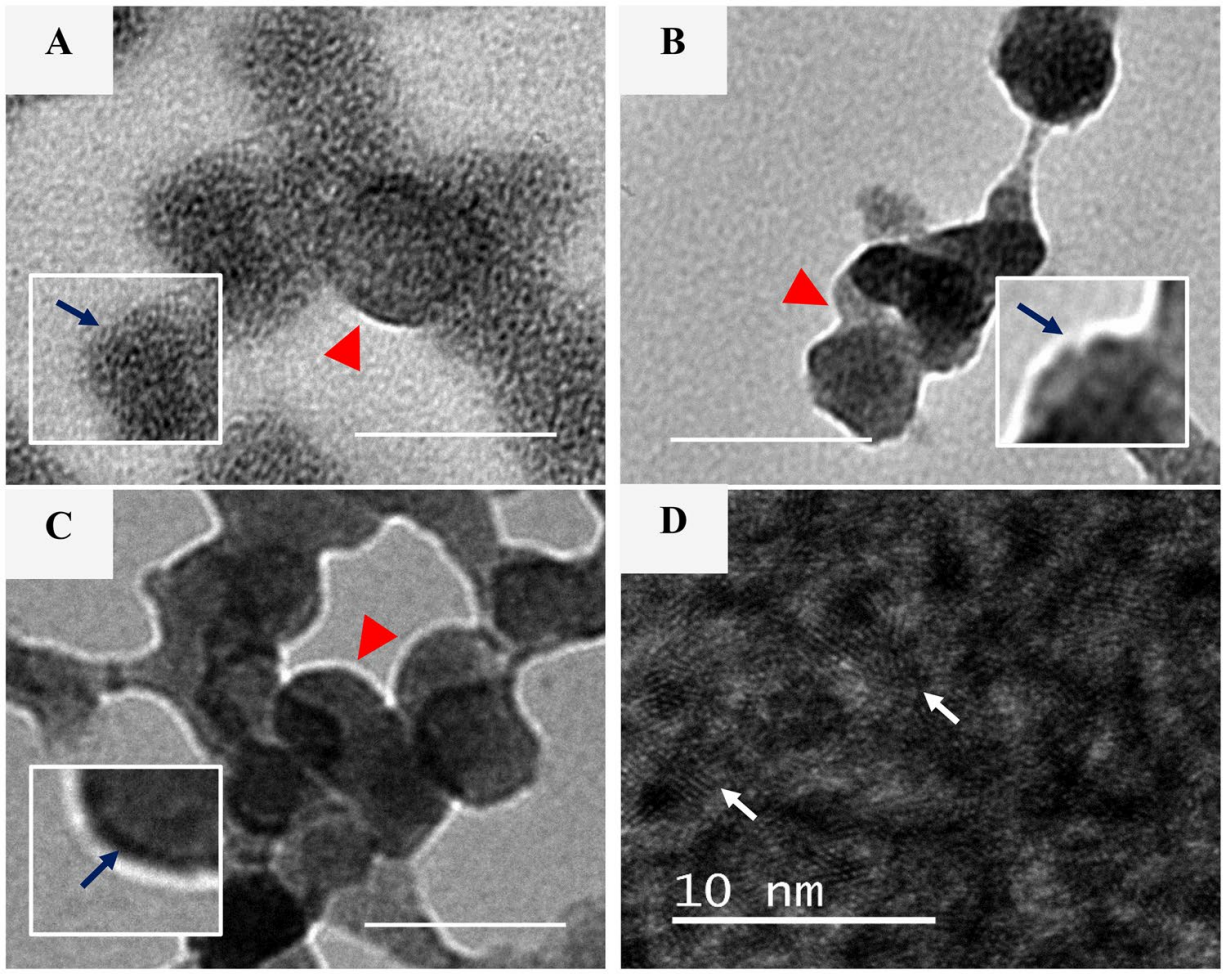
TEM images of pDNA-loaded lipid-Eudragit nanoparticles produced using **A)** microfluidics at FRR 3:1, **B)** microfluidics at FRR 5:1 and **C)** nanoprecipitation. Scale bar is 100 nm and images captured using 25,0000 × objective lens. **D)** High-magnification image of pDNA-loaded FRR 5:1 nanoparticles (400,000 × objective lens)

The morphology of lipid-Eudragit hybrid nanoparticles was assessed using high-resolution TEM. pDNA-loaded nanoparticles prepared using microfluidics generally displayed a spherical core-shell morphology (red arrow, Fig. 3A, B), similar to nanoparticles prepared using nanoprecipitation (Fig. 3C). A similar structure was obtained with unloaded nanoparticles (images not displayed). The hydrophobic Eudragit polymer is expected to form the core, enveloped by a lipid shell layer composed of DC-Cholesterol and DSPE-PEG [42]. The dark coloration observed on the nanoparticle surface was also a feature of the Eudragit S100 nanoparticle samples obtained by Kumar et al*.* [43].

The micrographs of nanoparticles prepared using nanoprecipitation and microfluidics indicated the presence of aggregates, with sizes corresponding to DLS measurements (Fig. 2). Ultra-high magnification of microfluidic samples showed a lattice network on the nanoparticle surface (white arrows, Fig. 3D), which is likely due to pDNA bound to the cationic lipid shell layer. Similar features were reported by Ripoll et al*.* [29] on lipid nanoparticles.

Closer examination of the lipid shell revealed structural differences between the nanoparticles (insets, Fig. 3A–C). Microfluidic FRR 3:1 nanoparticles displayed a morphologically damaged structure, with sparse coverage of the core by the lipid shell (blue arrow, Fig. 3A). FRR 5:1 nanoparticles showed a disrupted, discontinuous coverage of the polymer core (blue arrow, Fig. 3B). In contrast, nanoprecipitation samples displayed complete coverage of the core by the lipid shell (blue arrow, Fig. 3C).

These observed structural differences may be due to the prevalent flow conditions and mixing efficiency. The hybrid nanoparticles prepared using nanoprecipitation were formed by rapid, jet-wise addition of the organic phase to the aqueous phase featuring a central vortex pattern, induced by magnetic stirring (Fig. 1C). Hence, the flow conditions are expected to be turbulent in nature [44] and may be conducive to the formation of a complete lipid shell. In comparison, the bifurcating microfluidics toroidal design utilises laminar flow to form nanoparticles (Fig. 1B), whereby the organic and aqueous phases undergo splitting and recombining to achieve uniform mixing.

Ripoll et al. [29], captured the flow pattern in a bifurcating micromixer using a solution of alcohol and fluorescein. Their study indicated that FRR 3:1 (aqueous to organic phase) and TFR > 4 mL/min produced a highly uniform mixing regime and generated Reynolds numbers 220–1100, which are well below turbulence development regimes. The fluid dynamics existing in the bifurcating toroidal micromixer are highly complex and further complicated in the present study due to processing of non-Newtonian fluids. The observed nanoparticle morphology may be explained by a combination of factors, including the micromixer design, composition and concentration of the organic and aqueous phases and the presence of pDNA. These factors may influence the flow impedance and the subsequent fluid mixing characteristics within the micromixer due to viscosity changes [45]. Nevertheless, a key finding is that the resultant flow condition appears to disrupt lipid shell formation on the nanoparticle core of the microfluidic-prepared nanoparticles.

### DNA loading of nanoparticles

The application of microfluidics for nanoparticle production significantly improved pDNA encapsulation efficiency (90–99%) compared with nanoprecipitation (~73%). However, pDNA loading was not significantly different and was limited to 0.4% w/w for both preparation methods (Table 3).

**Table 3:** Encapsulation efficiency (EE) and pDNA loading of lipid-Eudragit hybrid nanoparticles prepared using microfluidics and nanoprecipitation

**Nanoparticle**	**Nanoprecipitation**	**Microfluidic flow rates**	
		3:1	5:1
**EE (%)**	73 ± 4	99 ± 1	90 ± 2
**Loading (% w/w)**	0.4 ± 1.0	0.4 ± 0.1	0.5 ± 0.1

These results demonstrate that lipid-Eudragit nanoparticles can achieve very high encapsulation efficiency but limited loading of pDNA, which is a typical finding of polymer and lipid-polymer hybrid nanoparticles encapsulating proteins and nucleic acids [35, 46]. For example, Dordelmann et al*.* [47] achieved 52% EE but 0.5% w/w loading of DNA in calcium phosphate/PLGA nanoparticles prepared using a double emulsion technique.

## Structural integrity of pDNA associated with nanoparticles

**Fig. 4:**
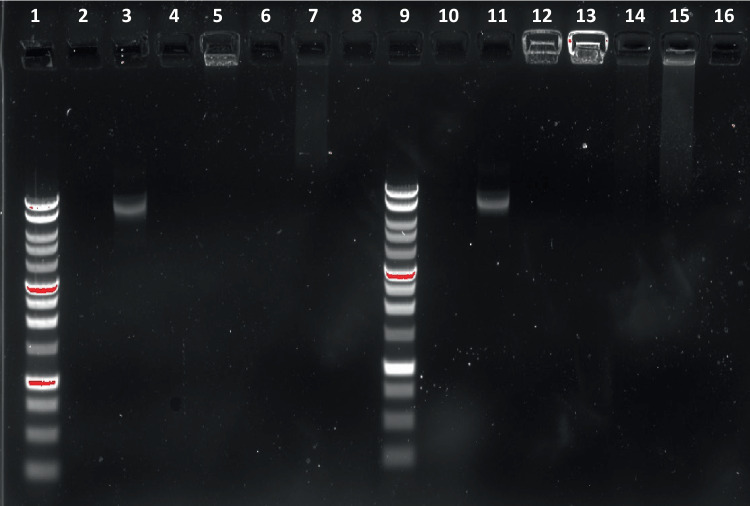
Gel electrophoresis imaging of untreated and heparin-treated lipid-Eudragit nanoparticles. Lane 1: 1 kB DNA ladder; 2: 1X TE buffer; 3: unformulated, ‘naked’ RFP pDNA; 4: unloaded FRR 5:1 nanoparticles; 5: pDNA-loaded FRR 5:1 nanoparticles; 6: heparin-treated unloaded FRR 5:1 nanoparticles; 7: heparin-treated pDNA-loaded FRR 5:1 nanoparticles; 8: heparin in 1X TE buffer (10 mg/mL); 9: 1 kB DNA ladder; 10: 1X TE buffer; 11: naked RFP pDNA; 12: unloaded FRR 3:1 nanoparticles; 13: pDNA-loaded FRR 3:1 nanoparticles; 14: heparin-treated unloaded FRR 3:1 nanoparticles; 15: heparin-treated pDNA-loaded FRR 3:1 nanoparticles; 16: heparin in 1X TE buffer (10 mg/mL)

Gel electrophoresis examination of microfluidic-prepared lipid-Eudragit nanoparticles identified tight binding of RFP pDNA to nanoparticles (lanes 5 and 13, Fig. 4). Similar observations were noted in nanoparticles produced using nanoprecipitation. Attempts to disrupt pDNA binding using high concentration heparin solution revealed only slight dissociation of pDNA (smear characteristic, lanes 7 and 15) and no band was visible. The observed fluorescence from unloaded 3:1 nanoparticles in lane 12 (Fig. 4) is due to association of the cyanine compound present in SYBR safe DNA stain with the DSPE-PEG surfactant present on the nanoparticle surface as reported previously [28].

The absence of degraded DNA species indicates that pDNA remained structurally intact throughout the formation and recovery stages of nanoparticle production. These findings are comparable with those of Wei et al*.* [26], who concluded from heparin treatment studies, that siRNA was completely protected in microfluidic PCL-PEI nanoparticles.

### Cytotoxicity of nanoparticles

**Fig. 5:**
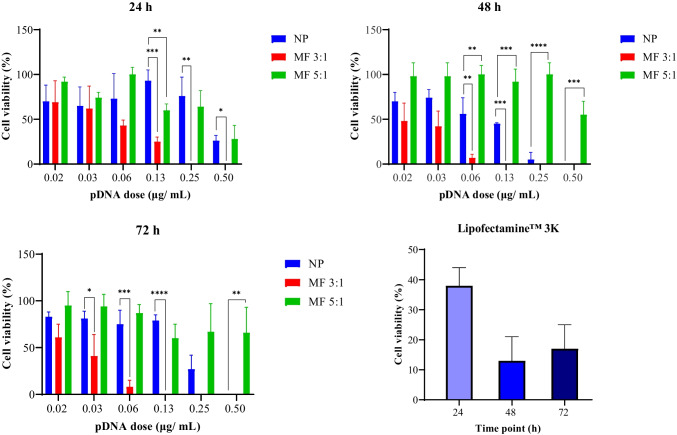
Cytotoxicity of pDNA-loaded lipid-Eudragit nanoparticles prepared using microfluidics at FRR 3:1, FRR 5:1, and nanoprecipitation, respectively (*n*=3 independent nanoparticle batches). MTT assay carried out at 24, 48, and 72 h, in line with transfection time points. Significance was determined using one-way ANOVA (^*^P: 0.01–0.05, ^**^P: 0.001–0.01, ^***^P: 0.0001–0.001, and ^****^P < 0.0001). Abbreviations: NP: nanoprecipitation; MF 3:1: microfluidics at FRR 3:1; MF 5:1: microfluidics at FRR 5:1. Commercial transfection reagent Lipofectamine™ 3K as positive control

The cytotoxicity of lipid-Eudragit hybrid nanoparticles was measured using the MTT assay following exposure of HEK293T cells to nanoparticles containing pDNA at increasing dose levels. Importantly, no flocculation of nanoparticles was observed during cytotoxicity and transfection assays, showing a resistance to ‘salting out’ in the presence of ionic species. Interestingly, the viability of HEK293T cells was extremely sensitive to the microfluidic flow conditions used to produce the nanoparticles, with FRR 3:1 samples producing higher cytotoxicity than FRR 5:1 samples (Fig. 5). Cell viability was also significantly different between microfluidics and nanoprecipitation-produced nanoparticles, with order of cytotoxicity as follows: FRR 3:1 > nanoprecipitation > FRR 5:1.

The biocompatibility of pDNA-loaded FRR 3:1 samples was poor (40–60%) at low pDNA concentrations (< 0.03 µg/mL) and decreased significantly (< 10% at 48–72 h) at 0.06 µg/mL. Zero cell viability was measured at pDNA concentration ≥ 0.25 µg/mL at all time points (Fig. 5). The commercial transfection reagent Lipofectamine™ 3000 delivering 1 µg/mL pDNA resulted in 23–36% cell viability at 24–72 h (Fig. 5), emphasizing the high cytotoxicity of the lipid-Eudragit nanoparticles prepared at FRR 3:1.

Nanoparticles produced at microfluidic FRR 5:1 resulted in higher cell viability at 0.02 to 0.50 µg/mL pDNA concentrations compared with nanoprecipitation samples (Fig. 5). More specifically, FRR 5:1 nanoparticles at low pDNA doses (0.02–0.06 µg/mL) maintained high cell viability of 80–100% compared with 50–80% for nanoparticles produced using nanoprecipitation. Complete cell death was measured at 0.50 µg/mL pDNA dose (48–72 h) for nanoprecipitation produced particles, whereas cell viability remained high (50–100%, 48–72 h) for microfluidic FRR 5:1 nanoparticles.

Similar results were obtained with unloaded nanoparticles (data not shown) whereas high cell viability (90–100%) was maintained with naked, unformulated pDNA. This behavior indicates that cell cytotoxicity arises from the nanoparticle carrier concentration rather than the pDNA content.

Based on pDNA loading for nanoprecipitation samples of 0.4% w/w and pDNA dose that inhibits cell growth by 50% (0.06 µg/mL), the calculated nanoparticle concentration is 14.9 µg/mL. Thus, the half maximal inhibitory concentration (IC_50_) of nanoprecipitation, microfluidic FRR 3:1, and FRR 5:1 samples is 14.9 µg/mL, 5.0 µg/mL, and 99.5 µg/mL respectively.

The high cytotoxicity of FRR 3:1 nanoparticles may be explained by their physicochemical characteristics described above. The positive surface charge of FRR 3:1 nanoparticles (+11 mV) was 2-fold higher than FRR 5:1 and nanoprecipitation particles respectively (+5 mV, Table 2), suggesting a reduced content of negatively charged DSPE-PEG surfactant on the surface of 3:1 nanoparticles (Table 1), which in turn may diminish ‘toxicity shielding effects’ associated with PEG [48]. Furthermore, TEM imaging of FRR 3:1 nanoparticles revealed sparse coverage of the lipid shell (Fig. 3A), which would be expected to increase cell exposure to the positively charged core. Hwang et al*.* [49] reported 0% viability of human neutrophil cells following exposure to PLGA or lipid nanoparticles coated with cationic CTAB surfactant and attributed the behaviour to the highly positive nanoparticles (+29–52 mV) interacting with the negatively charged cell membrane. The resulting Ca^2+^ influx was considered to trigger degranulation and cell death [49].

### *In vitro* transfection profile of lipid-Eudragit hybrid nanoparticles

Microfluidics is acknowledged to confer many benefits as a manufacturing platform for gene nanomedicines by way of realising complex designs with high reproducibility, continuous production, and control of nanoparticle size with uniform size distribution [14]. While these factors are definite attributes for industrial-scale processing, improvements in the biological performance of nanoparticles prepared using microfluidics compared with conventional techniques have not been unequivocally established [50]. Zoqlam et al*.* [31] found that nanoprecipitation of PLGA/Eudragit nanoparticles achieved 12% in vitro green fluorescent protein (GFP) transfection compared with 5% using microfluidics, although the authors attributed this characteristic to the higher DNA loading in nanoprecipitation samples. Huang et al*.* [51] identified greater down-regulation of vascular endothelial growth factor (VEGF) in vivo using microfluidic produced lipid-PEI nanoparticles (69%) than nanoprecipitation (44%).

**Fig. 6:**
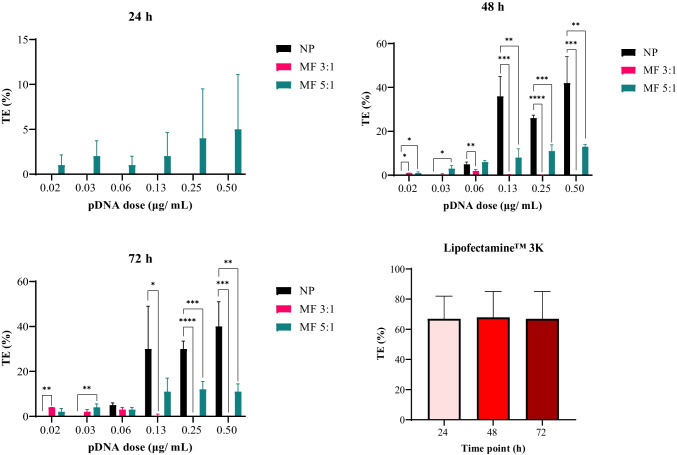
RFP expression in HEK293T cells following transfection with microfluidic and nanoprecipitation-prepared lipid-Eudragit hybrid nanoparticles, respectively (*n*=3 independent nanoparticle batches). Significance was determined using one-way ANOVA (^*^P: 0.01–0.05, ^**^P: 0.001–0.01, ^***^P: 0.0001–0.001, and ^****^P < 0.0001). Abbreviations: NP: nanoprecipitation; MF 3:1: microfluidics at FRR 3:1; MF 5:1: microfluidics at FRR 5:1. Commercial transfection reagent Lipofectamine™ 3K as positive control

**Fig. 7:**
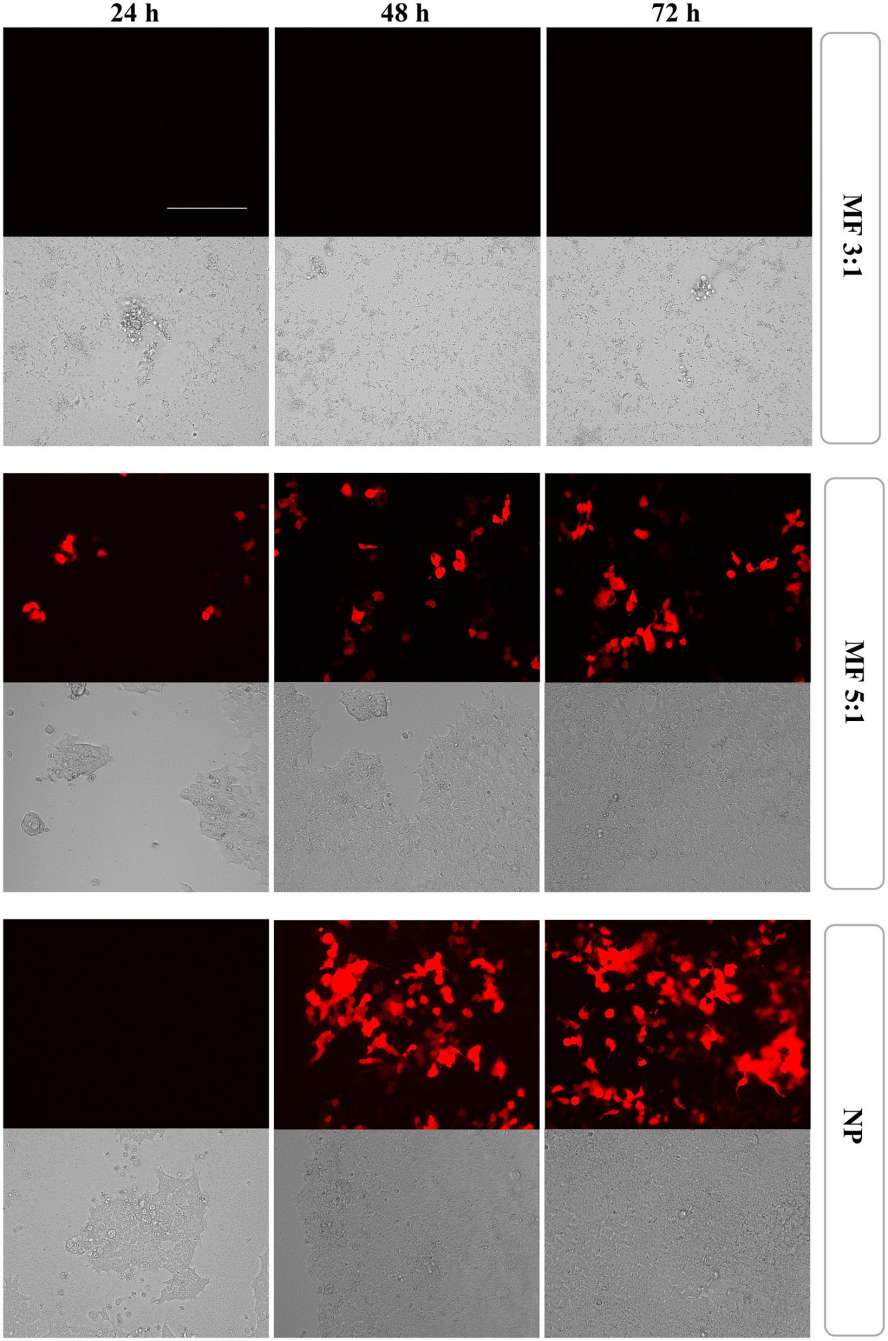
Representative transfection images of lipid-Eudragit hybrid nanoparticles produced using microfluidics and nanoprecipitation respectively. Images display transfection obtained at equivalent pDNA dose=0.25 µg/mL. Top panel displays RFP fluorescence images, bottom panel displays brightfield images. Scale bar is 150 µm. Abbreviations: NP: nanoprecipitation; MF 3:1: microfluidics at FRR 3:1; MF 5:1: microfluidics at FRR 5:1

In this work, the transfection efficiency (TE) of pDNA-loaded lipid-Eudragit nanoparticles prepared using microfluidics and nanoprecipitation, respectively, was examined at equivalent pDNA concentrations in HEK293T cells. While some reports indicate the poor reproducibility of conventional methods for nanoparticle preparation [14, 39], we found that the lipid-Eudragit hybrid nanoparticles achieved reproducible pDNA transfection whether prepared using microfluidics or nanoprecipitation (*n*=*3* independent batches). Similar to cytotoxicity findings, RFP expression was strongly dependent on the method of nanoparticle production and microfluidic flow conditions (Figs. 6 and 7). FRR 3:1 nanoparticles achieved low TE of 2 ± 1% at pDNA concentrations of 0.02–0.06 µg/ mL (Fig. 6), with no discernible transfection above 0.06 µg/mL, coinciding with findings of cell death at equivalent pDNA doses (Fig. 5). Delayed release of pDNA into the cell cytoplasm was indicated since transfection was measured at 48–72 h but was absent at 24 h.

Microfluidic FRR 5:1 nanoparticles, in comparison, resulted in a significant increase in transfection efficiency to 10–13% at 48–72h (Figs. 6 and 7), indicating gradual release of pDNA into the cell cytoplasm, due to pH-sensitive dissolution of Eudragit E100 polymer at endolysosomal pH=5. The positive control Lipofectamine™ 3000, achieved consistent RFP expression at high efficiency (65 ± 15%) at all time points (Fig. 6), indicating rapid pDNA transport into the cytoplasm and high transfection activity, characteristic of lipid-based systems.

The extreme sensitivity of nanoparticle biological properties to microfluidic flow conditions was also reported by Quagliarini et al*.* [27] for cationic lipid nanoparticles produced using a staggered herringbone design microfluidics mixer. Nanoparticles prepared at TFR=2 mL/min were 1–2 orders of magnitude more efficient in luciferase transfection in HEK293 cells than those prepared at TFR=8 mL/min. Microfluidic FRR 5:1 samples were notably less effective (TE 10–13%) than those produced using nanoprecipitation (TE 25–38%) at equivalent pDNA concentrations. This behavior may be connected with the disrupted lipid shell feature, evidenced by TEM (Fig. 3B) and the consequent reduced lipid presence at the nanoparticle surface, compared with those prepared using nanoprecipitation (Fig. 3C). Indeed, studies have shown that nanoparticles composed of Eudragit alone produce low in vitro DNA transfection (0–12%) [30–32].

## Conclusion

Microfluidics provides a platform for continuous production of nanomedicines on an industrial-scale. Lipid-polymer hybrid nanoparticles based on Eudragit E100 and containing pDNA were produced in a single-stage process using a bifurcating toroidal microfluidics mixer. Nanoparticle size and surface charge were similar to those produced using nanoprecipitation (size=150 nm, PDI=0.2, ξ-potential=5–11 mV). High-resolution TEM revealed the expected core-shell structure, but the lipid shell was disrupted in microfluidic samples. Nanoparticles produced at FRR 3:1 resulted in high toxicity towards HEK293T cells and low TE of 2%, while FRR 5:1 conditions resulted in significantly higher cell viability (50–100%) and TE of 12%. These findings provide an illustration of the potential of lipid-Eudragit hybrid nanoparticles as a carrier for gene medicines and highlights the importance of controlling microfluidics flow conditions to maximise their transfection capability.

## Data Availability

The authors confirm that the data supporting the findings of this study are available within the article. Raw, derived data supporting the findings of this study are available from first author, DS on request.

## References

[1] He W, Xing X, Wang X, Wu D, Wu W, Guo J, et al. Nanocarrier-mediated cytosolic delivery of biopharmaceuticals. Adv Func Mater. 2020;30(37):1910566.

[2] Li W, Qiu J, Li X-L, Aday S, Zhang J, Conley G, et al. BBB pathophysiology–independent delivery of siRNA in traumatic brain injury. Sci Adv. 2021;7(1):eabd6889. 10.1126/sciadv.abd6889 PMC7775748 33523853

[3] Abbas M, Baig MMFA, Zhang Y, Yang Y-S, Wu S, Hu Y, et al. A DNA-based nanocarrier for efficient cancer therapy. Journal of Pharmaceutical Analysis. 2021;11(3):330–9. 34277121 10.1016/j.jpha.2020.03.005 PMC8264464

[4] Li Z, Zhang X-Q, Ho W, Bai X, Jaijyan DK, Li F, et al. Lipid-polymer hybrid “particle-in-particle” nanostructure gene delivery platform explored for lyophilizable DNA and mRNA COVID-19 vaccines. Adv Func Mater. 2022;32(40):2204462. 10.1002/adfm.202204462 PMC9349454 35942271

[5] Schoenmaker L, Witzigmann D, Kulkarni JA, Verbeke R, Kersten G, Jiskoot W, et al. mRNA-lipid nanoparticle COVID-19 vaccines: structure and stability. Int J Pharm. 2021;601: 120586. 33839230 10.1016/j.ijpharm.2021.120586 PMC8032477

[6] Operti MC, Bernhardt A, Grimm S, Engel A, Figdor CG, Tagit O. PLGA-based nanomedicines manufacturing: technologies overview and challenges in industrial scale-up. Int J Pharm. 2021;605: 120807. 34144133 10.1016/j.ijpharm.2021.120807

[7] Piperno A, Sciortino MT, Giusto E, Montesi M, Panseri S, Scala A. Recent advances and challenges in gene delivery mediated by polyester-based nanoparticles. Int J Nanomedicine. 2021;16:5981–6002. 34511901 10.2147/IJN.S321329 PMC8418317

[8] Hadinoto K, Sundaresan A, Cheow WS. Lipid–polymer hybrid nanoparticles as a new generation therapeutic delivery platform: a review. Eur J Pharm Biopharm. 2013;85(3, Part A):427–43. 23872180 10.1016/j.ejpb.2013.07.002

[9] Mukherjee A, Waters AK, Kalyan P, Achrol AS, Kesari S, Yenugonda VM. Lipid-polymer hybrid nanoparticles as a next-generation drug delivery platform: state of the art, emerging technologies, and perspectives. Int J Nanomedicine. 2019;14:1937–52. 30936695 10.2147/IJN.S198353 PMC6430183

[10] Blanco E, Shen H, Ferrari M. Principles of nanoparticle design for overcoming biological barriers to drug delivery. Nat Biotechnol. 2015;33(9):941–51. 26348965 10.1038/nbt.3330 PMC4978509

[11] Su X, Fricke J, Kavanagh DG, Irvine DJ. In vitro and in vivo mRNA delivery using lipid-enveloped pH-responsive polymer nanoparticles. Mol Pharm. 2011;8(3):774–87. 21417235 10.1021/mp100390w PMC3354687

[12] Yang HW, Ye L, Guo XD, Yang C, Compans RW, Prausnitz MR. Ebola vaccination using a DNA vaccine coated on PLGA-PLL/γPGA nanoparticles administered using a microneedle patch. Adv Healthc Mater. 2017;6(1). 10.1002/adhm.201600750 28075069

[13] Yasar H, Biehl A, De Rossi C, Koch M, Murgia X, Loretz B, et al. Kinetics of mRNA delivery and protein translation in dendritic cells using lipid-coated PLGA nanoparticles. J Nanobiotechnology. 2018;16(1):72. 30231888 10.1186/s12951-018-0401-y PMC6145106

[14] Hamdallah SI, Zoqlam R, Erfle P, Blyth M, Alkilany AM, Dietzel A, et al. Microfluidics for pharmaceutical nanoparticle fabrication: the truth and the myth. Int J Pharm. 2020;584: 119408. 32407942 10.1016/j.ijpharm.2020.119408

[15] Abstiens K, Goepferich AM. Microfluidic manufacturing improves polydispersity of multicomponent polymeric nanoparticles. Journal of Drug Delivery Science and Technology. 2019;49:433–9.

[16] Shepherd SJ, Issadore D, Mitchell MJ. Microfluidic formulation of nanoparticles for biomedical applications. Biomaterials. 2021;274: 120826. 33965797 10.1016/j.biomaterials.2021.120826 PMC8752123

[17] Ahn J, Ko J, Lee S, Yu J, Kim Y, Jeon NL. Microfluidics in nanoparticle drug delivery; from synthesis to pre-clinical screening. Adv Drug Deliv Rev. 2018;128:29–53. 29626551 10.1016/j.addr.2018.04.001

[18] Liu Y, Yang G, Hui Y, Ranaweera S, Zhao C-X. Microfluidic nanoparticles for drug delivery. Small. 2022;18(36):2106580. 10.1002/smll.202106580 35396770

[19] Tomeh MA, Zhao X. Recent advances in microfluidics for the preparation of drug and gene delivery systems. Mol Pharm. 2020;17(12):4421–34. 33213144 10.1021/acs.molpharmaceut.0c00913

[20] Webb C, Forbes N, Roces CB, Anderluzzi G, Lou G, Abraham S, et al. Using microfluidics for scalable manufacturing of nanomedicines from bench to GMP: a case study using protein-loaded liposomes. Int J Pharm. 2020;582: 119266. 32251694 10.1016/j.ijpharm.2020.119266

[21] Bendre A, Bhat MP, Lee K-H, Altalhi T, Alruqi MA, Kurkuri M. Recent developments in microfluidic technology for synthesis and toxicity-efficiency studies of biomedical nanomaterials. Materials Today Advances. 2022;13: 100205.

[22] Valencia PM, Basto PA, Zhang L, Rhee M, Langer R, Farokhzad OC, et al. Single-step assembly of homogenous lipid-polymeric and lipid-quantum dot nanoparticles enabled by microfluidic rapid mixing. ACS Nano. 2010;4(3):1671–9. 20166699 10.1021/nn901433u PMC2923464

[23] Cerdá SL, Fontana F, Wang S, Correia A, Molinaro G, Tello RP, et al. Development of siRNA and budesonide dual-loaded hybrid lipid–polymer nanoparticles by microfluidics technology as a platform for dual drug delivery to macrophages: an in vitro mechanistic study. Advanced Therapeutics. 2023;6(8):2300048.

[24] Meyer RA, Hussmann GP, Peterson NC, Santos JL, Tuesca AD. A scalable and robust cationic lipid/polymer hybrid nanoparticle platform for mRNA delivery. Int J Pharm. 2022;611: 121314. 34838950 10.1016/j.ijpharm.2021.121314

[25] Rouco H, García-García P, Évora C, Díaz-Rodríguez P, Delgado A. Screening strategies for surface modification of lipid-polymer hybrid nanoparticles. Int J Pharm. 2022;624: 121973. 35811041 10.1016/j.ijpharm.2022.121973

[26] Wei W, Sun J, Guo X-Y, Chen X, Wang R, Qiu C, et al. Microfluidic-based holonomic constraints of siRNA in the kernel of lipid/polymer hybrid nanoassemblies for improving stable and safe in vivo delivery. ACS Appl Mater Interfaces. 2020;12(13):14839–54. 32182035 10.1021/acsami.9b22781

[27] Quagliarini E, Renzi S, Digiacomo L, Giulimondi F, Sartori B, Amenitsch H, et al. Microfluidic formulation of DNA-loaded multicomponent lipid nanoparticles for gene delivery. Pharmaceutics. 2021;13(8):1292. 34452253 10.3390/pharmaceutics13081292 PMC8400491

[28] Santhanes D, Wilkins A, Zhang H, John Aitken R, Liang M. Microfluidic formulation of lipid/polymer hybrid nanoparticles for plasmid DNA (pDNA) delivery. Int J Pharm. 2022;627: 122223. 36155792 10.1016/j.ijpharm.2022.122223

[29] Ripoll M, Martin E, Enot M, Robbe O, Rapisarda C, Nicolai M-C, et al. Optimal self-assembly of lipid nanoparticles (LNP) in a ring micromixer. Sci. 2022;12(1):9483. 10.1038/s41598-022-13112-5 PMC9177731 35676394

[30] Kanthamneni N, Yung B, Lee RJ. Effect of Eudragit on in vitro transfection efficiency of PEI-DNA complexes. Anticancer Res. 2016;36(1):81–5. 26722030

[31] Zoqlam R, Morris CJ, Akbar M, Alkilany AM, Hamdallah SI, Belton P, et al. Evaluation of the benefits of microfluidic-assisted preparation of polymeric nanoparticles for DNA delivery. Mater Sci Eng C Mater Biol Appl. 2021;127: 112243. 34225883 10.1016/j.msec.2021.112243

[32] Gargouri M, Sapin A, Bouli S, Becuwe P, Merlin JL, Maincent P. Optimization of a new non-viral vector for transfection: Eudragit nanoparticles for the delivery of a DNA plasmid. Technol Cancer Res Treat. 2009;8(6):433–44. 19925027 10.1177/153303460900800605

[33] Carugo D, Bottaro E, Owen J, Stride E, Nastruzzi C. Liposome production by microfluidics: potential and limiting factors. Sci. 2016;6(1):25876. 10.1038/srep25876 PMC4872163 27194474

[34] Gdowski A, Johnson K, Shah S, Gryczynski I, Vishwanatha J, Ranjan A. Optimization and scale up of microfluidic nanolipomer production method for preclinical and potential clinical trials. J Nanobiotechnology. 2018;16(1):12. 29433518 10.1186/s12951-018-0339-0 PMC5808420

[35] Roces CB, Christensen D, Perrie Y. Translating the fabrication of protein-loaded poly(lactic-co-glycolic acid) nanoparticles from bench to scale-independent production using microfluidics. Drug Deliv Transl Res. 2020;10(3):582–93. 31919746 10.1007/s13346-019-00699-y PMC7228990

[36] Solomun JI, Cinar G, Mapfumo P, Richter F, Moek E, Hausig F, et al. Solely aqueous formulation of hydrophobic cationic polymers for efficient gene delivery. Int J Pharm. 2021;593: 120080. 33246046 10.1016/j.ijpharm.2020.120080

[37] Li G, Huang T, Wang L, Gao Y. Methods and applications: Computational genomics: Frontiers Media SA; 2022.

[38] Dave V, Tak K, Sohgaura A, Gupta A, Sadhu V, Reddy KR. Lipid-polymer hybrid nanoparticles: synthesis strategies and biomedical applications. J Microbiol Methods. 2019;160:130–42. 30898602 10.1016/j.mimet.2019.03.017

[39] Streck S, Neumann H, Nielsen HM, Rades T, McDowell A. Comparison of bulk and microfluidics methods for the formulation of poly-lactic-co-glycolic acid (PLGA) nanoparticles modified with cell-penetrating peptides of different architectures. Int J Pharm X. 2019;1: 100030. 31517295 10.1016/j.ijpx.2019.100030 PMC6733288

[40] Fattal E, Fay F. Nanomedicine-based delivery strategies for nucleic acid gene inhibitors in inflammatory diseases. Adv Drug Deliv Rev. 2021;175: 113809. 34033819 10.1016/j.addr.2021.05.019

[41] Meireles M, Bourgeois F, Tourbin M, Guiraud P, Frances C. Review: removal of oversize & recovery of particles from suspensions in the nano size range. [Research Report] CNRS. 2010;hal-01186033.

[42] Aljabbari A, Lokras AG, Kirkensgaard JJK, Rades T, Franzyk H, Thakur A, et al. Elucidating the nanostructure of small interfering RNA-loaded lipidoid-polymer hybrid nanoparticles. J Colloid Interface Sci. 2023;633:907–22. 36508398 10.1016/j.jcis.2022.11.141

[43] Kumar N, Aggarwal R, Chauhan MK. Extended levobunolol release from Eudragit nanoparticle-laden contact lenses for glaucoma therapy. Future Journal of Pharmaceutical Sciences. 2020;6(1):109.

[44] Fessi H, Puisieux F, Devissaguet JP, Ammoury N, Benita S. Nanocapsule formation by interfacial polymer deposition following solvent displacement. Int J Pharm. 1989;55(1):R1–4.

[45] Wild A, Taylor R, inventors, The University of British Columbia , Vancouver, Canada, assignee. Bifurcating mixers and methods of their use and manufacture. Canada patent US 10, 076, 730 B2. 2018 Sep18.

[46] Astete CE, Sabliov CM. Synthesis and characterization of PLGA nanoparticles. J Biomater Sci Polym Ed. 2006;17(3):247–89. 16689015 10.1163/156856206775997322

[47] Dordelmann G, Kozlova D, Karczewski S, Lizio R, Knauer S, Epple M. Calcium phosphate increases the encapsulation efficiency of hydrophilic drugs (proteins, nucleic acids) into poly(d, l-lactide-co-glycolide acid) nanoparticles for intracellular delivery. Journal of Materials Chemistry B. 2014;2(41):7250–9. 32261804 10.1039/c4tb00922c

[48] Lechanteur A, Furst T, Evrard B, Delvenne P, Hubert P, Piel G. PEGylation of lipoplexes: the right balance between cytotoxicity and siRNA effectiveness. Eur J Pharm Sci. 2016;93:493–503. 27593989 10.1016/j.ejps.2016.08.058

[49] Hwang TL, Aljuffali IA, Lin CF, Chang YT, Fang JY. Cationic additives in nanosystems activate cytotoxicity and inflammatory response of human neutrophils: lipid nanoparticles versus polymeric nanoparticles. Int J Nanomedicine. 2015;10:371–85. 25609950 10.2147/IJN.S73017 PMC4294622

[50] Ottonelli I, Duskey JT, Rinaldi A, Grazioli MV, Parmeggiani I, Vandelli MA, et al. Microfluidic technology for the production of hybrid nanomedicines. Pharmaceutics. 2021;13(9):1495. 34575571 10.3390/pharmaceutics13091495 PMC8465086

[51] Huang X, Lee RJ, Qi Y, Li Y, Lu J, Meng Q, et al. Microfluidic hydrodynamic focusing synthesis of polymer-lipid nanoparticles for siRNA delivery. Oncotarget. 2017;8(57):96826–36. 29228574 10.18632/oncotarget.18281 PMC5722526

